# Imaging analysis and predictive nomogram construction for degenerative lumbar spondylolisthesis with severe clinical symptom based on propensity score matching

**DOI:** 10.1038/s41598-023-31224-4

**Published:** 2023-03-13

**Authors:** Yi He, Wei Wang, Haiting Zhou, Xiaojian Huang, Yinguang Wang, Yunkun Qu, Hao Cheng, Hongbo You

**Affiliations:** 1 Department of Orthopedics, Tongji Hospital, Tongji Medical College, Huazhong University of Science and Technology, Wuhan, 430030 People’s Republic of China; 2 Department of Oncology, Tongji Hospital, Tongji Medical College, Huazhong University of Science and Technology, Wuhan, 430030 People’s Republic of China

**Keywords:** Diseases, Health care, Risk factors

## Abstract

Intervertebral disc degeneration, local lumbar segmental morphology changes, and atrophy of multifidus muscle have been considered to be associated with degenerative lumbar spondylolisthesis. However, there remains a great deal of controversy. To further investigate their relationship with degenerative lumbar spondylolisthesis, we conducted a retrospective study that included 67 patients with degenerative spondylolisthesis and 182 control subjects. Propensity score matching was employed to match the case group and the control group. Disc height was evaluated by the anterior disc height index (DHIA) and posterior disc height index (DHIP). Local lumbar segmental morphology was assessed by segmental lordosis (SL). The fatty infiltration and atrophy of multifidus muscle was evaluated by multifidus muscle net content (MFNC). Our results indicate that DHIA, DHIP, SL, and MFNC in the case group were significantly lower than in the control group. Furthermore, the DHIA, DHIP, and MFNC of the slipped segment (L4/5) were lower than those of the non-slipped segment (L3/4). Correlation analysis showed a high relationship between DHIA and MFNC and the degree of degenerative lumbar spondylolisthesis. Logistic regression analysis revealed that DHIA and MFNC might act as protective factors against the development of degenerative lumbar spondylolisthesis. Additionally, a prognostic nomogram was developed and validated to assess the likelihood of patients with severe symptoms requiring surgical intervention.

## Introduction

Lumbar spondylolisthesis is one of the most common causes of low back pain^1,2^. It can be divided into several types, including isthmic, traumatic, degenerative, pathologic, dysplastic, and postsurgical spondylolisthesis^3^. Of these, degenerative spondylolisthesis is the most common subtype^4^. The incidence of degenerative lumbar spondylolisthesis is approximately 6%, affecting individuals over 50, with a male-to-female ratio of 1:2–1:6, typically involving the L4-L5 segment^2,5–8^. The degree of slippage is usually mild, with an average of 14%^6^. While most patients present with mild symptoms that can be managed conservatively, those with persistent severe low back pain often require surgical intervention^1,9^. The prevalence of degenerative lumbar spondylolisthesis is rising as the aging population grows, posing a significant economic burden^10–12^. Therefore, a comprehensive investigation of the risk factors associated with degenerative lumbar spondylolisthesis is critical for alleviating patient suffering while lessening the social care burden.

The etiology of degenerative lumbar spondylolisthesis is multifaceted and complex. It is commonly acknowledged that advanced age, female sex (particularly postmenopausal women with reduced estrogen levels), and obesity are significant risk factors for degenerative lumbar spondylolisthesis^8,13–15^. Moreover, alterations in the local structure and morphology of the lumbar spine also represent noteworthy factors in the etiology of degenerative lumbar spondylolisthesis. Current research findings consistently indicate that disc degeneration constitutes a significant association with degenerative lumbar spondylolisthesis and was an independent risk factor^16–19^. Furthermore, spinal-pelvic parameters exert a substantial impact on lumbar spinal stability, whereby sacral slope (SS), pelvic incidence (PI), and lumbar lordosis (LL) were found to be related to degenerative lumbar spondylolisthesis and positively associated with the extent of slippage^20–22^. Some researchers have also investigated the role of paraspinal muscle and reported a potential association between the quality of these muscles and lumbar spondylolisthesis^23,24^. Nevertheless, the etiology of degenerative lumbar spondylolisthesis is still not fully understood, necessitating further investigation.

Propensity score matching (PSM) is a statistical technique that can effectively mitigate confounding variables, allowing for a more accurate comparison between case and control groups. As such, this study employed propensity score matching to address potential bias related to age and gender differences between cases and controls. We aimed to investigate the association between lumbar intervertebral disc degeneration, segmental lordosis, and fatty infiltration and atrophy of the multifidus muscle with degenerative lumbar spondylolisthesis using both X-ray and magnetic resonance imaging (MRI). Furthermore, we sought to construct a nomogram capable of predicting the likelihood of patients with severe symptoms who may require surgical intervention.

## Result

### Propensity score matching

**Figure 1:**
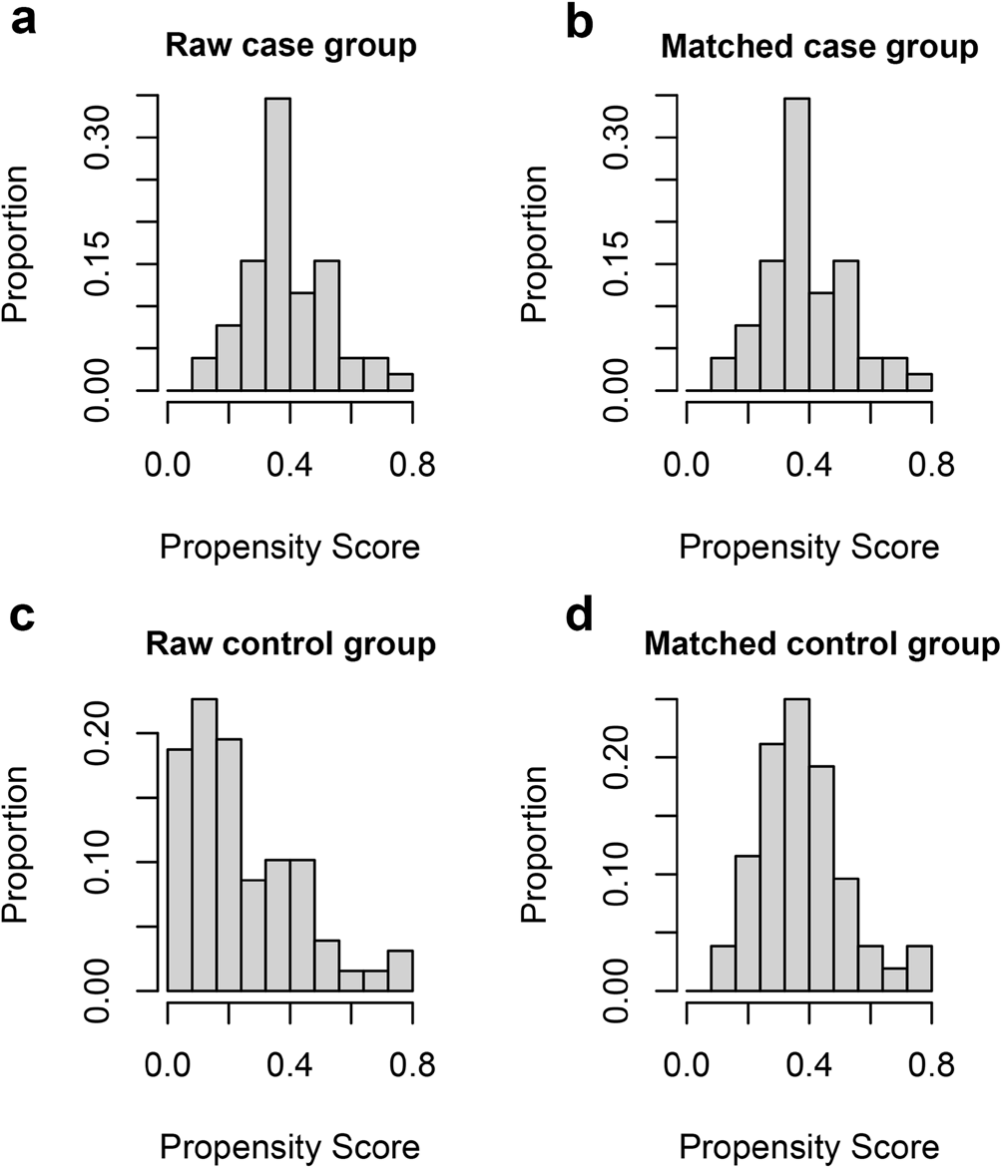
Propensity score matching results. (**a**) Distribution of propensity scores in the raw case group. (**b**) Distribution of propensity scores in the matched case group. (**c**) Distribution of propensity scores in the raw control group. (**d**) Distribution of propensity scores in the matched control group.

Prior to propensity matching, there were apparent differences in propensity scores between the control group and the case group, indicating differences in age and gender between the two groups. After propensity score matching, there were 50 patients in the case group and 50 in the control group (Table 1), and the distribution of propensity scores was similar between the two groups (Fig. 1a–d). In the case group, there were 14 male and 36 female participants, with an average age of 57.92 ± 7.42. The control group consisted of 15 male and 35 female participants, with an average age of 58.12 ± 7.36. The matching of the two groups was verified using the chi-square test and t-test, which revealed no statistically significant difference in terms of gender and age (*P* = 1.000 and 0.893, respectively) (Table 1).

**Table 1:** Age and gender information in the two groups after propensity score matching.

	Case	Control
N	50	50
Male (%)	14 (28.0%)	15 (30.0%)
Age (Mean ± SD)	57.92 ± 7.42	58.12 ± 7.36

### Consistency test

**Figure 2:**
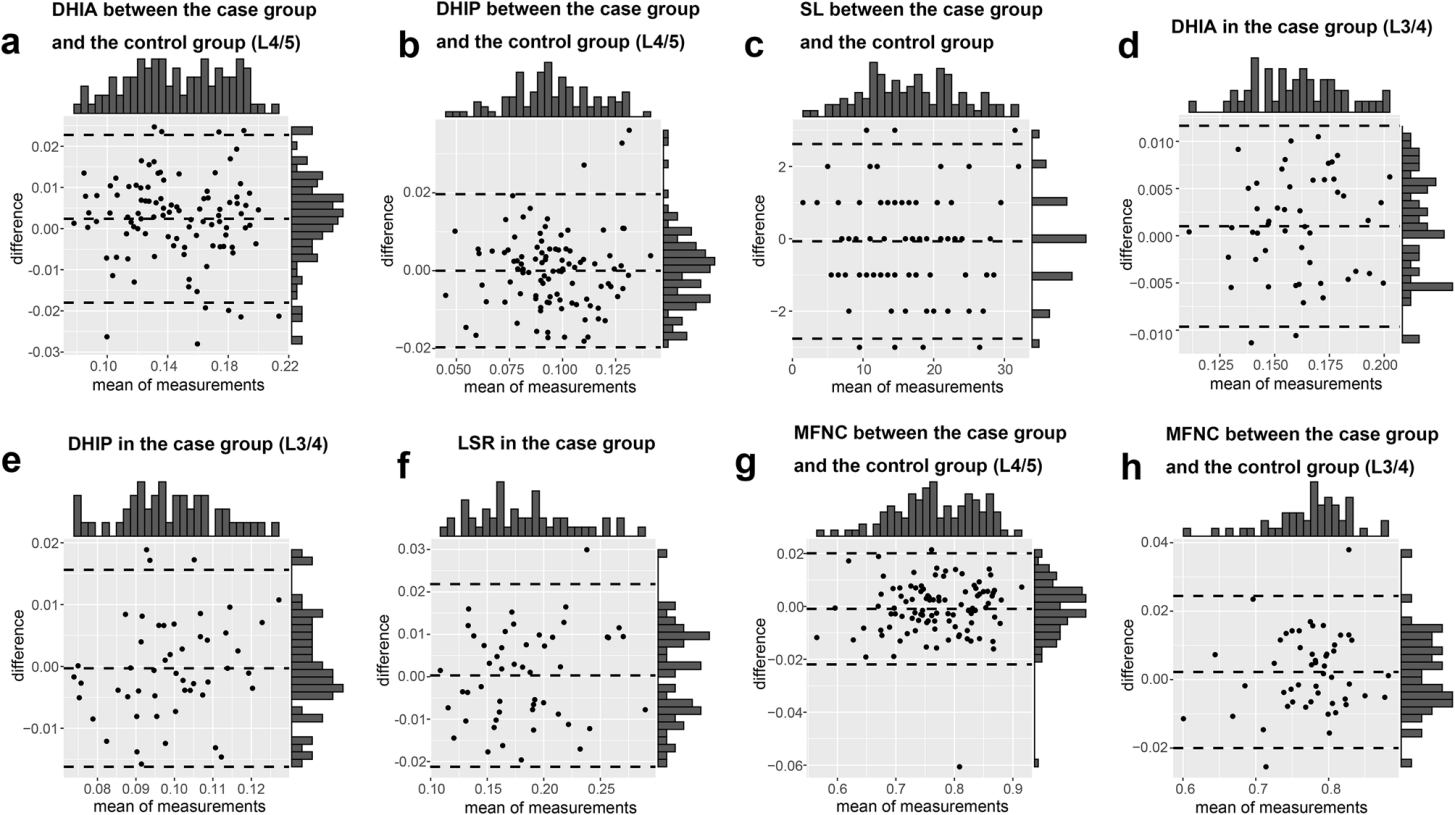
Bland–Altman plots for testing the consistency of data measured by two researchers. (**a**) DHIA between the case group and the control group (L4/5). (**b**) DHIP between the case group and the control group (L4/5). (**c**) SL between the case group and the control group. (**d**) DHIA in the case group (L3/4). (**e**) DHIP in the case group (L3/4). (**f**) LSR in the case group. (**g**) MFNC between the case group and the control group (L4/5). (**h**) MFNC between the case group and the control group (L3/4).

The intraclass correlation coefficients (ICCs) for all parameters surpassed 0.8 (Table 2), and the Bland–Altman plots exhibited that nearly all data points fell within the boundaries of the dotted line, signifying a high degree of concordance between the measurements conducted by the two researchers (Fig. 2a–h). The result of the Shapiro–Wilk test indicated the normal distribution of all data (Table 3). The average values were used for subsequent data analysis.

**Table 2:** ICC test.

Parameter	ICC	95% CI	
		Lower	Upper
DHIA between the case group and the control group (L4/5)	0.948	0.923	0.964
DHIP between the case group and the control group (L4/5)	0.881	0.828	0.918
DHIA in the case group (L3/4)	0.965	0.939	0.980
DHIP in the case group (L3/4)	0.821	0.706	0.894
SL between the case group and the control group	0.980	0.970	0.986
MFNC between the case group and the control group (L4/5)	0.989	0.983	0.992
MFNC between the case group and the control group (L3/4)	0.978	0.962	0.988
LSR in the case group	0.969	0.946	0.982

**Table 3:** The average values of each parameter and normality test.

Parameter	Mean ± SD	W	*P* value
DHIA in the case group (L4/5)	12.162% ± 2.325%	0.977	0.434
DHIA in the case group (L3/4)	16.061% ± 2.038%	0.989	0.908
DHIA in the control group (L4/5)	16.944% ± 2.015%	0.975	0.358
DHIP in the case group (L4/5)	8.493% ± 1.618%	0.977	0.433
DHIP in the case group (L3/4)	9.890% ± 1.285%	0.986	0.813
DHIP in the control group (L4/5)	10.613% ± 1.744%	0.982	0.646
SL in the case group	14.830° ± 6.660°	0.964	0.126
SL in the control group	18.820° ± 6.277°	0.980	0.564
MFNC in the case group (L4/5)	72.160% ± 5.571%	0.976	0.382
MFNC in the case group (L3/4)	77.534% ± 5.125%	0.969	0.203
MFNC in the control group (L4/5)	81.608% ± 4.856%	0.963	0.116

W: Indicator to judge the normal distribution. When W is close to 1 and P is more significant than 0.05, the data can be considered to obey the normal distribution.

### Comparison between the case group and the control group

The homogeneity of variance was confirmed for DHIA, DHIP, SL, and MFNC by variance test (*P* > 0.05). Subsequently, an independent sample t-test was conducted, revealing significant differences in DHIA, DHIP, SL, and MFNC between the case and control groups (*P* < 0.05) (Table 4).

**Table 4:** Comparison between the case group and the control group.

	Variance test		Independent sample t-test				
	F	*P*	t	Df	*P*	95% CI	
DHIA	0.223	0.638	− 10.991	98	< 0.001	− 5.645	− 3.918
DHIP	1.063	0.305	− 6.300	98	< 0.001	− 2.788	− 1.452
SL	0.001	0.971	− 3.083	98	0.003	− 6.558	− 1.422
MFNC	0.545	0.462	− 9.041	98	< 0.001	− 11.522	− 7.374

*CI* Confidence interval.

### Comparison between slipped segment (L4/5) and non-slipped segment (L3/4) in the case group

The variance test confirmed the homogeneity of variance for DHIA, DHIP, and MFNC (*P* > 0.05). A paired sample t-test was subsequently conducted, revealing significant differences in DHIA, DHIP, and MFNC between the slipped (L4/5) and non-slipped (L3/4) segments (*P* < 0.05) (Table 5).

**Table 5:** Comparison between slipped segment (L4/5) and non-slipped segment (L3/4) in the case group.

	Variance test		Paired sample t-test					
	F	*P*	Mean	t	Df	*P*	95% CI	
DHIA	0.255	0.615	− 3.898	− 11.946	49	< 0.001	− 4.554	− 3.243
DHIP	1.281	0.261	− 1.396	− 6.250	49	< 0.001	− 1.845	− 0.947
MFNC	0.378	0.540	− 5.374	− 11.549	49	< 0.001	− 6.310	− 4.439

*CI* Confidence interval.

### Correlation analysis

**Figure 3:**
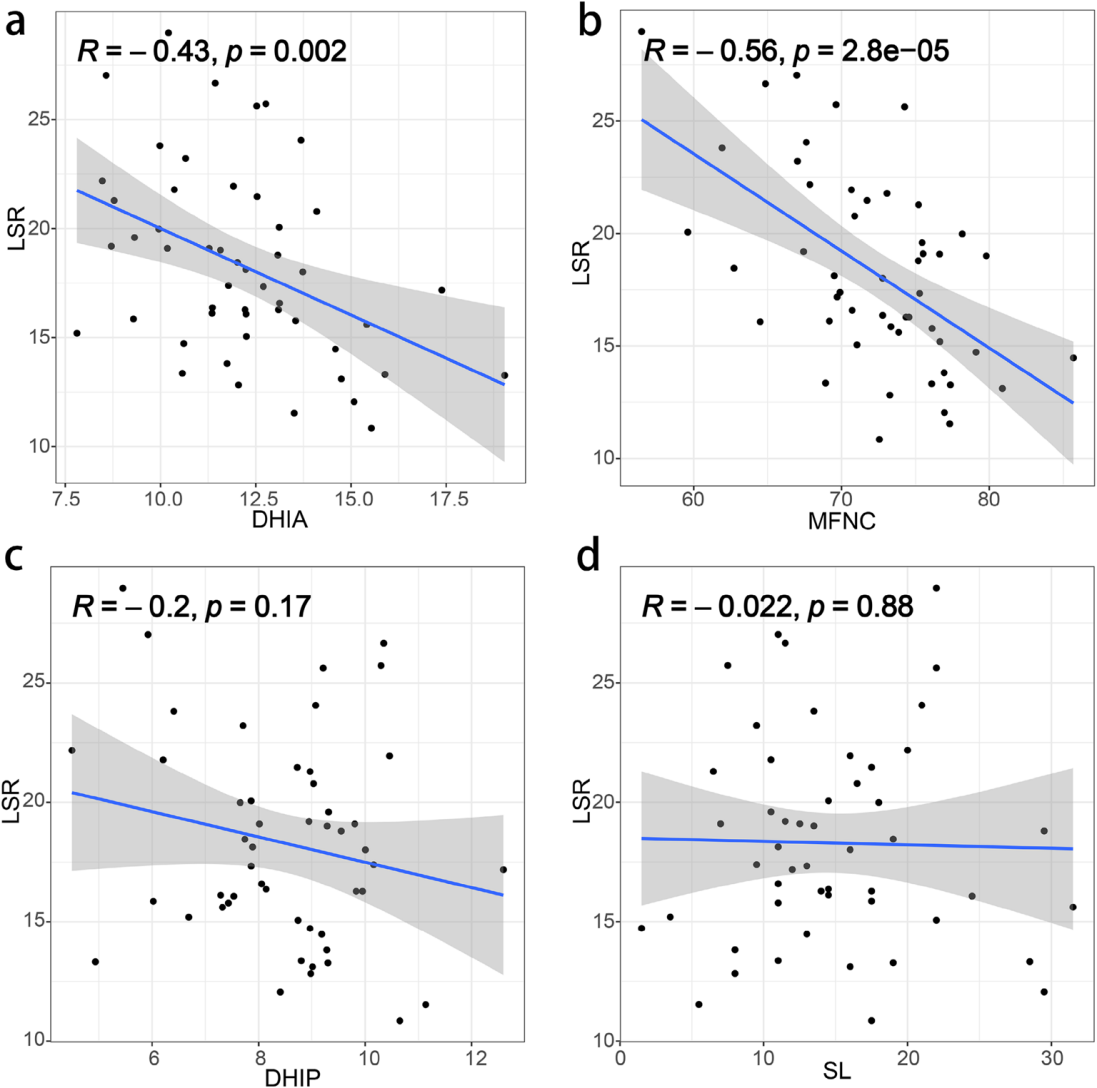
Correlation analysis showed that LSR was negatively correlated with the DHIA (**a**) and the MFNC (**b**), while there were no correlations between LSR and DHIP (**c**) and SL (**d**).

To begin with, we conducted a correlation analysis between LSR and several morphological parameters (DHIA, DHIP, SL, MFNC). Our findings revealed that LSR exhibited a significant negative correlation with DHIA (R = − 0.430, *P* = 0.002) (Fig. 3a) and MFNC (R = − 0.560, *P* < 0.001) (Fig. 3b), while no significant correlation was observed between LSR and DHIP (R = − 0.200, *P* = 0.170) (Fig. 3c) or SL (R = − 0.022, *P* = 0.880) (Fig. 3d).

**Figure 4:**
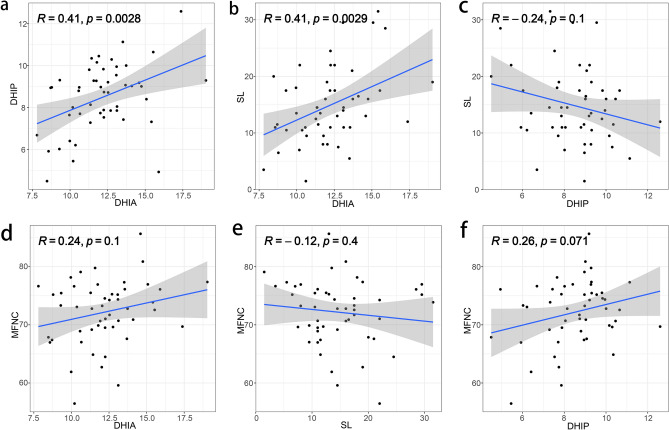
The correlation analysis showed that the DHIA positively correlated with the DHIP (**a**) and SL (**b**). Other correlations were not observed (**c**–**f**).

Thereafter, an analysis was conducted to examine the correlations among DHIA, DHIP, SL, and MFNC. The findings revealed a positive correlation between DHIA and DHIP (R = 0.410, *P* = 0.0028) (Fig. 4a) as well as between DHIA and SL (R = 0.410, *P* = 0.0029) (Fig. 4b), while no other significant correlations were observed (*P* > 0.05) (Fig. 4c–f).

### Logistic regression

The findings of the stepwise logistic regression analysis revealed that DHIA (OR = 0.470, *P* < 0.001) and MFNC (OR = 0.731, *P* < 0.001) exhibited a protective effect against the development of degenerative spondylolisthesis (Table 6). This also suggested that a decrease in anterior disc height and fatty infiltration and atrophy of the multifidus muscle were potential risk factors for degenerative spondylolisthesis.

**Table 6:** Logistic regression.

	OR	95% CI		*P*
		Lower	Upper	
DHIA	0.470	0.291	0.672	< 0.001
MFNC	0.731	0.589	0.859	< 0.001

*CI* Confidence interval.

### Nomogram

**Figure 5:**
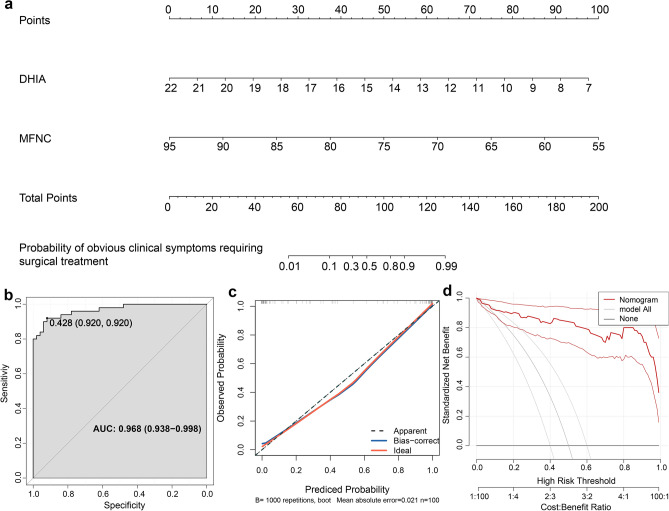
The development of a nomogram for predicting the likelihood of patients with severe symptoms requiring surgical intervention. (**a**) Details of the nomogram. (**b**) The receiver operating characteristic (ROC) curve indicated excellent discrimination by the nomogram. (**c**) The calibration curve exhibited strong agreement between the predicted and observed outcomes. (**d**) The decision curve analysis (DCA) curve exhibited a higher benefit for patients across a broad spectrum of predicted probabilities.

Based on logistic regression analysis results, a prognostic model was established to predict the likelihood of patients with severe symptoms requiring surgical intervention. Through vertical alignment to the 'Point' row, each measured parameter yielded a corresponding point aggregated to obtain a total point. Using this total point, we could infer the likelihood of a patient's symptoms deteriorating and necessitating surgical intervention. Most patients in our study had scores ranging from 0 to 200 (Fig. 5a). The nomogram demonstrated favorable discrimination, evidenced by an AUC of 0.968 (95% confidence interval = 0.938–0.998) (Fig. 5b). The calibration curves of the nomogram showed high consistency between the predicted and observed outcomes (Fig. 5c). Internal validation using fivefold cross-validation, Jackknife validation, and Bootstrap validation yielded mean AUC values of 0.965, 0.958, and 0.968, respectively, as well as mean C-index values of 0.966, 0.958, and 0.968, respectively (Table 7). These results provided strong evidence of accuracy of the nomogram. Decision curve analysis (DCA) also demonstrated that the nomogram exhibited a higher benefit for patients across a broad spectrum of predicted probabilities (Fig. 5d). In conclusion, the nomogram exhibited considerable discriminative and calibrating abilities.

**Table 7:** Internal validation of the nomogram.

Validation methods	Mean ROC	Mean C-index	Iteration times	Failed iterations
fivefold cross-validation	0.965	0.966	200	0
Jackknife validation	0.958	0.958	100	0
Bootstrap validation	0.968	0.968	200	0

## Discussion

It is widely acknowledged that gender and age are risk factors for degenerative lumbar spondylolisthesis. Moreover, local instability of the lumbar spine may also contribute to its development. Current research suggests that disc degeneration is the underlying cause of degenerative lumbar spondylolisthesis. Anderson et al. examined lumbar spine radiographs of 304 patients with degenerative lumbar spondylolisthesis and found that the mean disc height was 7 mm^25^. In contrast, Bach et al. discovered that the mean disc height at the L4/5 segment was 8.9 mm ± 1.7 mm in healthy men and 8.6 mm ± 1.8 mm in healthy women^26^, indicating that disc height was reduced to some extent in patients with degenerative lumbar spondylolisthesis. Additionally, Aono et al. conducted a prospective study in which they followed 142 women without lumbar spondylolisthesis for an average of 12 years and found that L4 degenerative lumbar spondylolisthesis was significantly associated with a reduction in anterior disc height of L4/5^21^. Chen et al. also found that disc height was substantially smaller in patients with degenerative lumbar spondylolisthesis than in the control group and that reduced anterior disc height was an independent predictor^17^. Our study found that DHIA and DHIP were significantly lower in the case group, especially the DHIA, which decreased to a greater extent, indicating that loss of disc height, particularly anterior disc height, was associated with degenerative lumbar spondylolisthesis. Furthermore, similar results were observed between slipped segment (L4/5) and the non-slipped segment (L3/4) in the case group. These findings were consistent with previous studies. Further correlation analysis revealed a negative correlation between DHIA and the LSR, while no correlation existed between DHIP and LSR. Logistic regression analysis revealed that only DHIA was a protective factor for degenerative lumbar spondylolisthesis. Based on these results, we speculated that although there was a simultaneous loss of anterior disc height and posterior disc height in patients with degenerative lumbar spondylolisthesis, anterior disc height loss was predominant and might have a more significant influence on posterior disc height loss.

Spinal-pelvic parameters play an important role in spinal balance. SL is the angulation between the superior endplates of the upper segment and the inferior endplates of the lower segment, which could reflect the morphology of the local lumbar spine. Although previous studies have examined the effect of SL on lumbar spine surgery, its relationship with degenerative lumbar spondylolisthesis has been less explored^27–29^. Recently, the French Spine Society and Kepler et al. proposed the French classification^30,31^ and CARDS classification^32^ for degenerative lumbar spondylolisthesis, respectively, both of which include the degree of preservation of SL as a classification criterion. These classifications suggest that a decrease in SL, or even a change to negative values, indicates an unstable local lumbar spine prone to slippage progression. In our study, we observed a significant difference in SL between the case and control groups, with the case group exhibiting a significantly smaller SL than the control group. Correlation analysis revealed that SL was only associated with DHIA. However, logistic regression analysis showed that SL was neither a protective nor risk factor for degenerative lumbar spondylolisthesis. Based on previous findings that degenerative lumbar spondylolisthesis was associated with a more significant loss of anterior than posterior disc height, we speculated that the imbalance of anterior and posterior disc height loss might cause the change in SL. Further research is required to understand better the pathological mechanisms underlying changes in SL in patients with degenerative lumbar spondylolisthesis.

Hiyama A et al. found that skeletal muscle mass was associated with spinal-pelvic parameters, especially pelvic tilt^33^. The multifidus muscle is the skeletal muscle closest to the spine, playing a crucial role in controlling segmental motion and maintaining the stability of the lumbar spine^34^. Multifidus muscle degeneration, characterized by the decreased cross-sectional area (CSA) and increased fat infiltration, has been linked to low back pain^35,36^. Wallwork et al. compared the size and muscle activity of the multifidus muscle at L4 in 34 subjects. They found that the CSA of the multifidus muscle was significantly smaller in those with chronic pain than in those without^37^. Similarly, Goubert et al. assessed muscle structure characteristics and muscle activity for 55 adults with non-specific low back pain and found greater fatty infiltration of the multifidus muscle in those with continuous chronic low back pain^34^. Since low back pain is a prominent symptom of degenerative lumbar spondylolisthesis, there might be a relationship between multifidus muscle degeneration and degenerative lumbar spondylolisthesis. In our study, we found that MFNC was significantly reduced in the case group, and that MFNC was negatively correlated with the degree of spondylolisthesis. Logistic regression analysis also indicated that MFNC was a protective factor in degenerative lumbar spondylolisthesis. These results also suggested that fatty infiltration and atrophy of the multifidus muscle was associated with degenerative lumbar spondylolisthesis and was a risk factor. Other studies have reported similar results. Wang et al. found that the degree of atrophy of the multifidus muscle was significantly greater in degenerative lumbar spondylolisthesis patients^23^. Guo et al. found that CSA of the multifidus muscle was significantly smaller in patients with degenerative lumbar spondylolisthesis than people in the control group with lumbar disc herniation or lumbar spinal stenosis^38^. Fatty infiltration and atrophy of multifidus muscle might be associated with muscle disuse. It has been shown that after 60 days of bed rest, subjects showed significant atrophy of the multifidus muscle at the L4 and L5, which was highly correlated with low back pain. Furthermore, the reduction in CSA of the multifidus muscle was significantly greater than that of erector spinae muscle in healthy individuals who were chronically bedridden^39^. The paravertebral muscle in adult individuals undergoes progressive degeneration over time, with the multifidus muscle being particularly susceptible to this process^40^. Kjaer et al. also found that women were more likely than men to have fatty infiltration of the multifidus muscle^41^. Hiyama et al. suggested that increasing skeletal muscle mass through nutritional guidance and muscle strengthening exercise might help to reduce pain in patients with spinal disorders^33^. O’Sullivan et al. found that subjects who underwent a 10-week specific exercise treatment program involving the specific training of the deep abdominal muscles, with co-activation of the lumbar multifidus muscle, had a statistically significant reduction in pain intensity and functional disability levels^42^. Hides et al. also revealed that patients, who received specific exercise, experienced fewer recurrences of low back pain than those in the control group^43^. Based on our findings, we supposed that the elderly or females might have less daily exercise and activity compared to the youngers or males, which made the paravertebral muscles, especially the multifidus muscle, more prone to fatty infiltration, disuse atrophy, and consequent weakening of the strength of the multifidus muscle. This could make the local lumbar spine unstable and increase the risk of lumbar spondylolisthesis. Therefore, appropriate functional exercises for the paravertebral muscles, especially the multifidus muscle, are recommended for the elderly and females, which might play a crucial role in preventing low back pain.

Given that most patients diagnosed with degenerative lumbar spondylolisthesis exhibit mild clinical symptoms that can be relieved through conservative treatment, some of them may still experience disease progression and a worsening of clinical symptoms, it is imperative to forecast the likelihood of symptom exacerbation in affected individuals accurately. As a result, we developed a nomogram capable of estimating the probability of patients experiencing severe symptoms necessitating surgical intervention. If the nomogram predicts a high probability of severe clinical symptoms, proactive implementation of physical therapy and other recommended interventions should be prioritized to forestall the progression of degenerative spondylolisthesis.

However, this study also has limitations. Foremost, due to the study’s retrospective nature, causality between the identified factors and the development of degenerative lumbar spondylolisthesis cannot be definitively established. There might be other confounding factors that we have not taken into account, such as the association of low back pain itself with fatty infiltration of the muscle. Furthermore, the sample size following propensity score matching was relatively modest. As a result, future prospective investigations employing larger sample sizes are warranted to elucidate these findings further.

## Conclusion

The reduction of disc height, particularly in the anterior region, as well as the decrease of SL and the infiltration of fat and atrophy of the multifidus muscle, were observed to be more pronounced in patients suffering from degenerative lumbar spondylolisthesis compared to both their non-slipped segments and the general population. These findings suggest that the loss of anterior disc height, as well as fat infiltration and multifidus muscle atrophy, may serve as potential risk factors for the development of degenerative spondylolisthesis. Furthermore, we developed a nomogram to assess the likelihood of patients with severe symptoms requiring surgical intervention.

## Methods

### Inclusion and exclusion criteria

**Figure 6:**
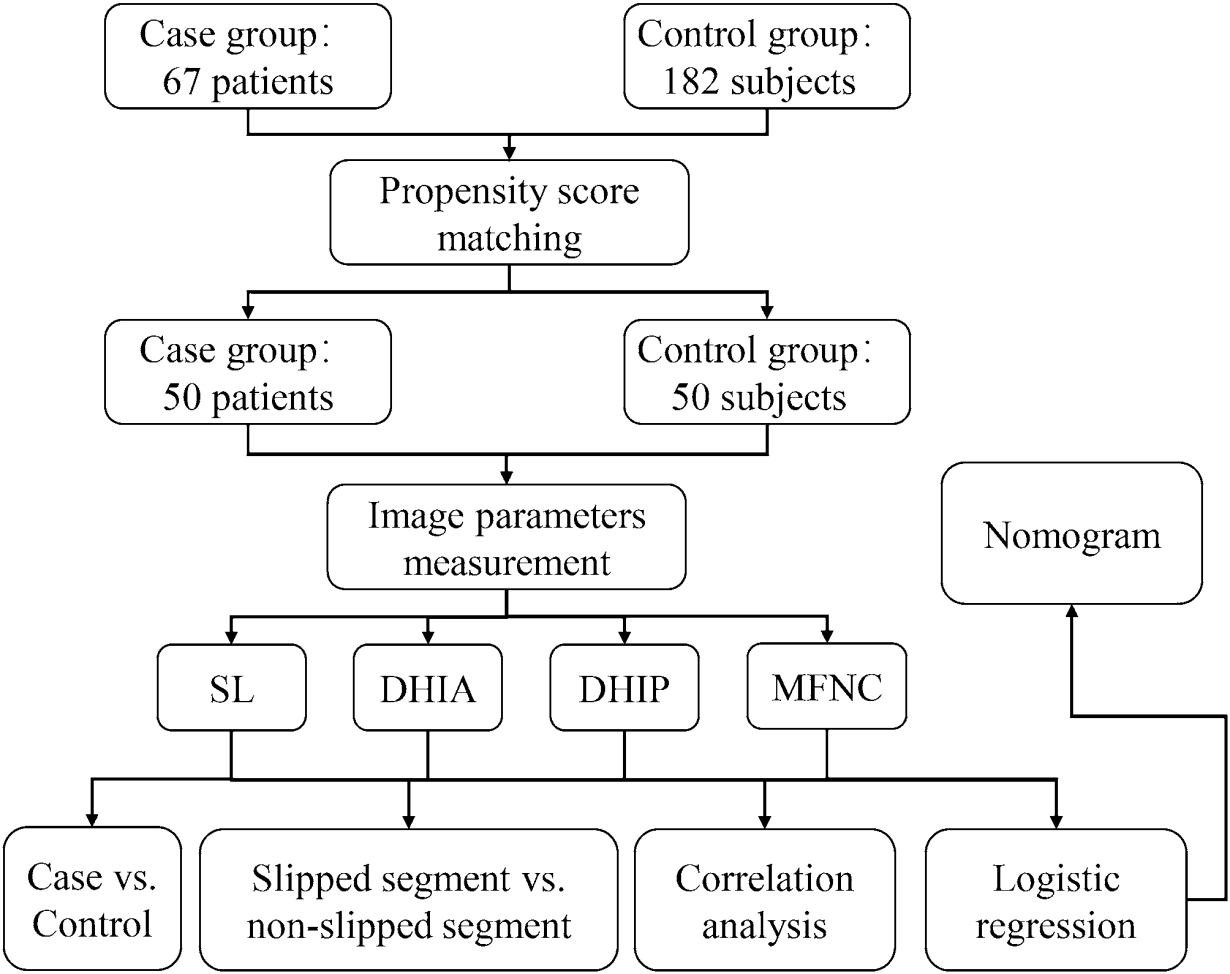
The workflow of our study.

This is a retrospective study. We collected clinical data, lumbar spine MRI, and X-ray data from a cohort of 67 patients with L4 degenerative spondylolisthesis who exhibited symptoms severe enough to warrant surgical intervention, as well as 182 asymptomatic outpatients between January 2016 and July 2020 at Tongji Hospital, Tongji Medical College, Huazhong University of Science and Technology. The case group was selected based on the following inclusion criteria: (1) patients underwent anteroposterior and lateral lumbar standing X-ray radiographs and MRI examinations; (2) patients were diagnosed with L4 degenerative lumbar spondylolisthesis. The exclusion criteria were as follows: (1) presence of multiple segmental lumbar spondylolisthesis, (2) diagnosis of isthmic spondylolisthesis, (3) presence of retrolisthesis, (4) presence of other conditions such as deformity, trauma, infection, tumor, or previous lumbar surgery. Meanwhile, the inclusion criteria for the control group were as follows: (1) patients who received anteroposterior and lateral lumbar standing X-ray radiographs and MRI examination, and (2) patients who had no clinical symptoms or only had low back pain, and imaging data excluded lumbar spondylolisthesis. Exclusion criteria for the control group included: (1) lumbar spondylolisthesis and (2) conditions such as deformity, trauma, infection, tumor, and previous lumbar surgery. This study was approved by the Ethics Committee of Tongji Hospital, Tongji Medical College, Huazhong University of Science and Technology (TJ-IRB20210501). Informed consent was exempted by Ethics Committee of Tongji Hospital, Tongji Medical College, Huazhong University of Science and Technology (TJ-IRB20210501). And here is the workflow of our study (Fig. 6).

### Propensity score matching

Based on the epidemiological investigation results, it was observed that the predilection for degenerative spondylolisthesis was among older women. However, as comparisons between the case and control groups could be confounded by differences in gender and age, their conclusions were subject to limited certainty. To address this issue, the present study utilized propensity score matching (PSM) to establish a well-balanced case and control groups. The nearest neighbor method in the “MatchIt” package of R software (version 3.6.2) was employed to calculate the propensity scores, considering both the gender and age of each patient. Subsequently, the propensity scores were employed to match the two groups of patients on a 1:1 ratio with an error rate of less than 0.02 (caliper value = 0.02). Finally, the “tableone” package was employed to validate the results of the matching procedure.

### Image evaluation

All individuals underwent anteroposterior and lateral lumbar X-ray radiography, as well as magnetic resonance imaging (MRI). Film viewing and parameter measurements were performed using the Picture Archiving and Communication System (PACS), specifically Fujifilm's Synapse Workstation (Version 3.2.1).

### Evaluation of intervertebral disc degeneration and slippage

**Figure 7:**
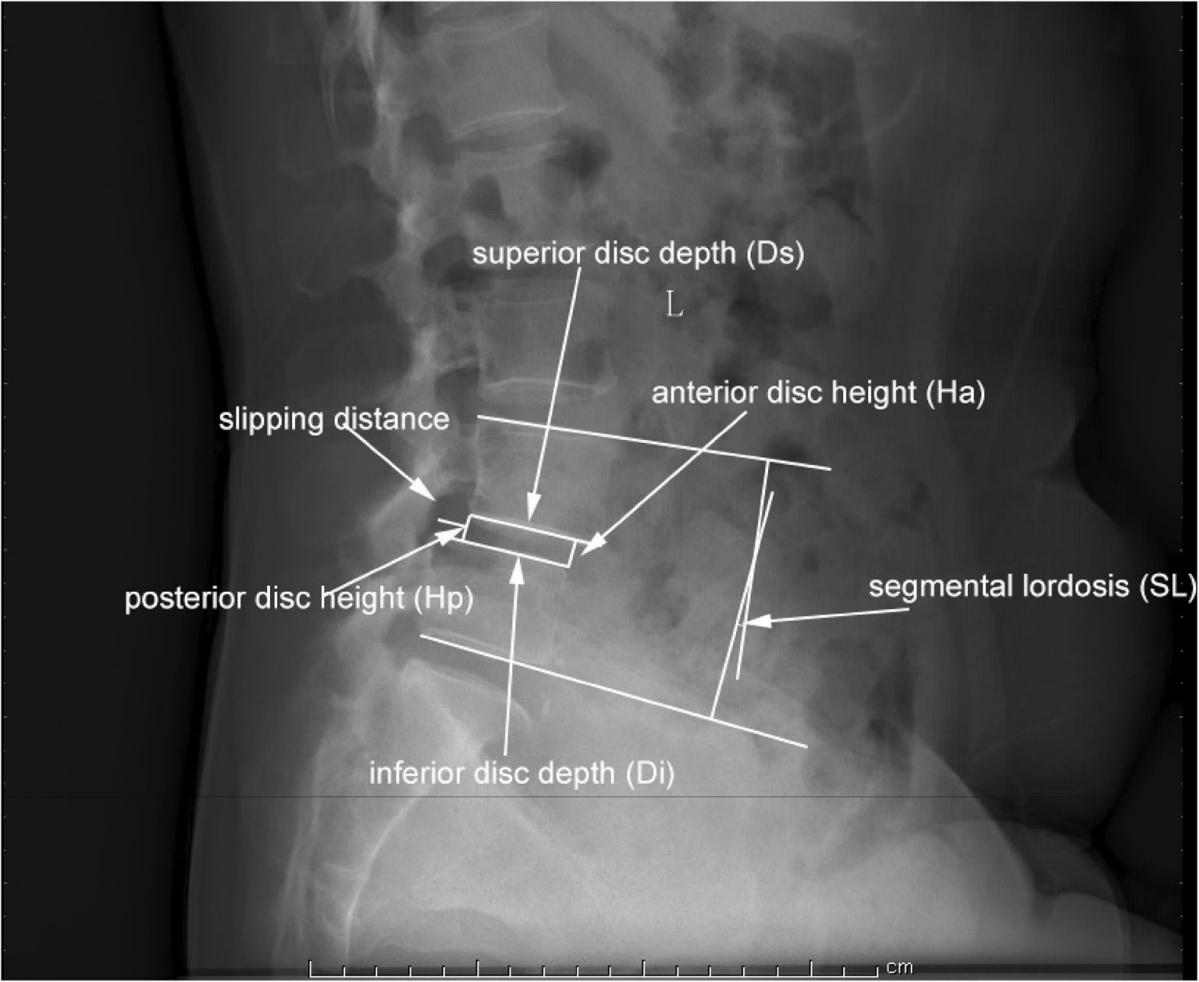
Parameters measured on X-ray radiographs. Anterior disc height index (DHIA) = [Ha/(Ds + Di)] *100%. Posterior disc height index (DHIP) = [Hp/(Ds + Di)] *100%.

On the lateral radiographs of the lumbar spine, we assessed several parameters, including anterior disc height (Ha), posterior disc height (Hp), superior disc depth (Ds), and inferior disc depth (Di) (Fig. 7). To evaluate disc height, we utilized the anterior disc height index (DHIA) and posterior disc height index (DHIP) based on established methodologies found in relevant literature^44,45^. Specifically, we calculated DHIA as [Ha / (Ds + Di)] *100% and DHIP as [Hp/ (Ds + Di)] *100%. In the case group, we analyzed these parameters separately for the slipped segment (L4/5) and the non-slipped segment (L3/4). For the control group, we evaluated these parameters in the L4/5 segment. It is important to note that smaller DHIA or DHIP values indicate more severe disc degeneration.

### Evaluation of the degree of lumbar spondylolisthesis

In the case group, we quantified the degree of lumbar spondylolisthesis by measuring the displacement between the superior and inferior vertebral bodies. This displacement was expressed as the lumbar spondylolisthesis ratio (LSR), calculated as (slipped distance/Di) *100%.

### Segmental lordosis

On the lateral radiographs of the lumbar spine, we assessed the segmental lordosis (SL) by measuring the angle between the superior endplates of L4 and the inferior endplates of L5. This measurement serves as an indicator of the local lumbar spine morphology.

### Evaluation of multifidus muscle

**Figure 8:**
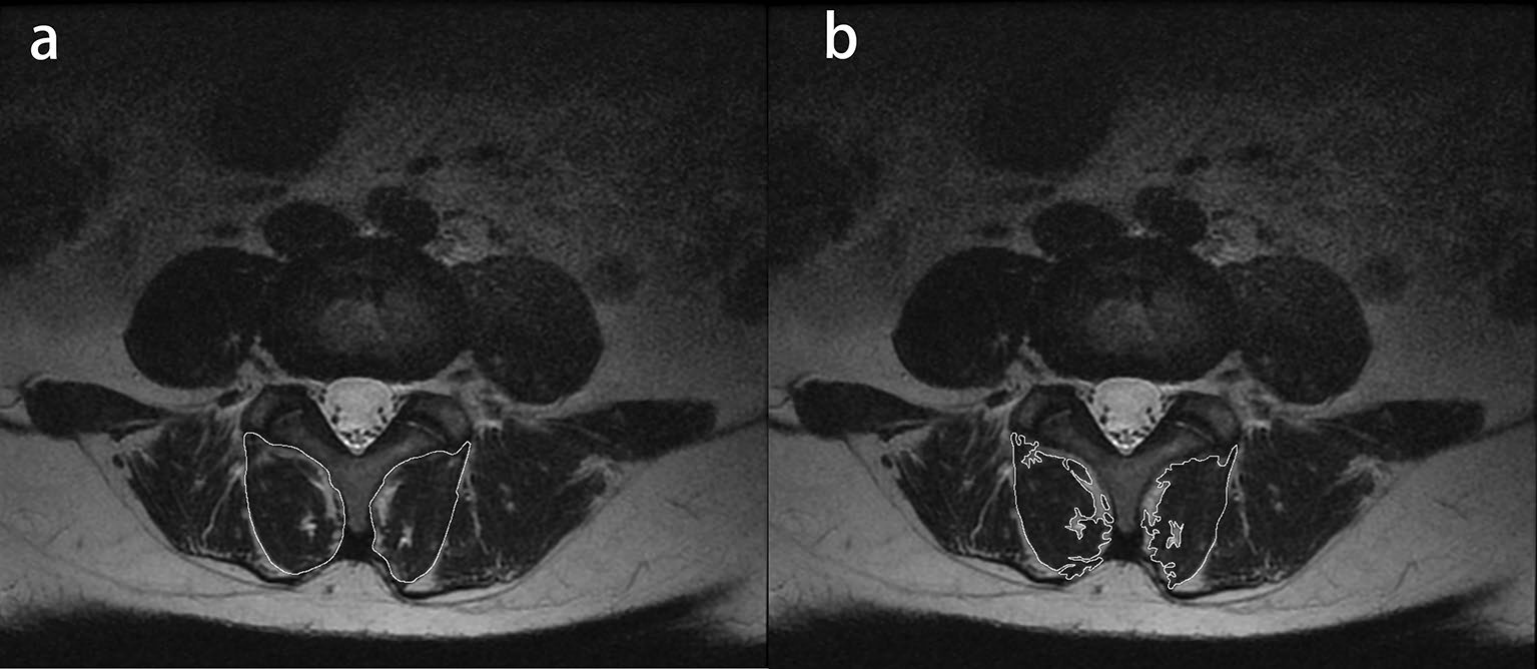
Parameters measured on MRI. (**a**) Total multifidus muscle cross-sectional area (TCSA). (**b**) Fat-free multifidus muscle cross-sectional area (FCSA).

The present study evaluated multifidus muscle morphology using T-2 weighted lumbar spine MRI. Total multifidus muscle cross-sectional area (TCSA) (Fig. 8a) and fat-free multifidus muscle cross-sectional area (FCSA) (Fig. 8b) were measured to assess muscle morphology. Fatty infiltration and atrophy of multifidus muscle were evaluated using multifidus muscle net content (MFNC), calculated as (FCSA/TCSA) *100%. In the case group, the above parameters were measured separately for the slipped segment (L4/5) and non-slipped segment (L3/4), while in the control group, the parameters were measured in the L4/5 segment. A lower MFNC value indicated a greater degree of multifidus atrophy.

### Consistency test

The parameters were independently measured and calculated by two researchers, and the mean values were utilized for subsequent statistical analysis by another researcher. The three researchers were blind to each other. Microsoft Excel 2019 was employed for data input and storage, while R software (V3.6.2) and related R program packages were used for data analysis. The “irr” package was employed to evaluate the intraclass correlation coefficient (ICC)^46^ for data consistency between the two researchers. When ICC (1) > 0.800, the results of the two researchers were deemed consistent. The “BlandAltmanLeh” package was also utilized to evaluate the data consistency of the two researchers’ measurements.

### Comparison of the parameters between the control group and the case group

Before comparing, the “Shapiro function” of the “car” package was employed to conduct the Shapiro–Wilk test, which ascertains whether the data was normally distributed. The variance test was performed using the R software base package's “leveneTest function”. Based on the results of the Shapiro–Wilk test and variance test, we performed either an independent sample t-test or Wilcoxon rank-sum test to compare the case group and the control group. *P* < 0.05 was considered statistically significant.

### Comparison of the parameters between the slipped segment (L4/5) and non-slipped segment (L3/4) in the case group

Similarly, we first test whether the data follow a normal distribution and whether the variances were heterogeneous. We then used a paired t-test or Wilcoxon rank-sum test to compare the slipped segment (L4/5) and non-slipped segment (L3/4). *P* < 0.05 was considered statistically significant.

### Correlation analysis

Pearson correlation analysis was carried out by using the “limma” package^47^, and the correlation scatter plots were graphed using “ggplot2”, “ggpubr”, and “ggExtra” packages. *P* < 0.05 was considered statistically significant.

### Logistic regression

Logistic regression was employed to analyze risk and protective factors for degenerative spondylolisthesis, and the odds ratios (OR) were calculated. *P* < 0.05 was considered statistically significant.

### Nomogram

In order to predict the probability of patients with severe symptoms requiring surgical intervention, we constructed a predictive nomogram using the “nomogram” package, which was based on the results of logistic regression. We then internally validated the model using fivefold cross-validation, Jackknife validation, and bootstrap validation, with 200 iterations for fivefold cross-validation and bootstrap validation, and 100 iterations for Jackknife validation. *P* < 0.05 was considered statistically significant.

### Ethics approval and consent to participate

This is a retrospective study, and the data collected and analyzed was the imaging data of the patients. According to the requirements of the ethics committee, informed consent can be exempted from a retrospective study. As a result, we made an application and received written approval from the Ethics Committee of Tongji Hospital, Tongji Medical College, Huazhong University of Science and Technology (Ethical approval number: TJ-IRB20210501). Informed consent was exempted by Ethics Committee of Tongji Hospital, Tongji Medical College, Huazhong University of Science and Technology (TJ-IRB20210501). All methods were performed in accordance with the relevant guidelines and regulations.

## Data Availability

The datasets generated during and analyzed during the current study are available from the corresponding author upon reasonable request.

## References

[1] Kalichman L (2009). Spondylolysis and spondylolisthesis: Prevalence and association with low back pain in the adult community-based population. Spine.

[2] Fredrickson BE, Baker D, McHolick WJ, Yuan HA, Lubicky JP (1984). The natural history of spondylolysis and spondylolisthesis. J. Bone Jt. Surg. Am..

[3] Tenny, S. & Gillis, C. C. in *StatPearls* (StatPearls Publishing Copyright © 2022, StatPearls Publishing LLC., 2022).

[4] Wiltse LL (1962). The etiology of spondylolisthesis. J. Bone Jt. Surg. Am..

[5] Frymoyer JW (1994). Degenerative spondylolisthesis: Diagnosis and treatment. J. Am. Acad. Orthop. Surg..

[6] Rosenberg NJ (1975). Degenerative spondylolisthesis. Predisposing factors. J. Bone Jt. Surg. Am..

[7] Koreckij TD, Fischgrund JS (2015). Degenerative spondylolisthesis. J. Spinal Disord. Tech..

[8] Jacobsen S, Sonne-Holm S, Rovsing H, Monrad H, Gebuhr P (2007). Degenerative lumbar spondylolisthesis: An epidemiological perspective: The Copenhagen Osteoarthritis Study. Spine.

[9] Lenz M (2022). Clinical outcome after lumbar spinal fusion surgery in degenerative spondylolisthesis: A 3-year follow-up. Arch. Orthop. Trauma Surg..

[10] Martin BI (2019). Trends in lumbar fusion procedure rates and associated hospital costs for degenerative spinal diseases in the United States, 2004 to 2015. Spine.

[11] Martin BI (2009). Trends in health care expenditures, utilization, and health status among US adults with spine problems, 1997–2006. Spine.

[12] Dagenais S, Caro J, Haldeman S (2008). A systematic review of low back pain cost of illness studies in the United States and internationally. Spine J..

[13] Kalichman L, Guermazi A, Li L, Hunter DJ (2009). Association between age, sex, BMI and CT-evaluated spinal degeneration features. J. Back Musculoskelet. Rehabil..

[14] Imada K, Matsui H, Tsuji H (1995). Oophorectomy predisposes to degenerative spondylolisthesis. J. Bone Jt. Surg. Br..

[15] Schuller S, Charles YP, Steib JP (2011). Sagittal spinopelvic alignment and body mass index in patients with degenerative spondylolisthesis. Eur. Spine J..

[16] Bydon M, Alvi MA, Goyal A (2019). Degenerative lumbar spondylolisthesis: Definition, natural history, conservative management, and surgical treatment. Neurosurg. Clin. N. Am..

[17] Chen IR, Wei TS (2009). Disc height and lumbar index as independent predictors of degenerative spondylolisthesis in middle-aged women with low back pain. Spine.

[18] Teichtahl AJ (2015). A Dose-response relationship between severity of disc degeneration and intervertebral disc height in the lumbosacral spine. Arthritis Res. Ther..

[19] Takashima H (2014). Investigation of intervertebral disc and facet joint in lumbar spondylolisthesis using T2 mapping. Magn. Reson. Med. Sci..

[20] Lai Q (2018). Correlation between the sagittal spinopelvic alignment and degenerative lumbar spondylolisthesis: A retrospective study. BMC Musculoskelet. Disord..

[21] Aono K, Kobayashi T, Jimbo S, Atsuta Y, Matsuno T (2010). Radiographic analysis of newly developed degenerative spondylolisthesis in a mean twelve-year prospective study. Spine.

[22] Nakamae T, Nakanishi K, Kamei N, Adachi N (2019). The correlation between sagittal spinopelvic alignment and degree of lumbar degenerative spondylolisthesis. J. Orthop. Sci..

[23] Wang G (2015). Quantitative MRI and X-ray analysis of disc degeneration and paraspinal muscle changes in degenerative spondylolisthesis. J. Back Musculoskelet. Rehabil..

[24] Park JH (2019). Association of MRI-defined lumbar paraspinal muscle mass and slip percentage in degenerative and isthmic spondylolisthesis: A multicenter, retrospective, observational study. Medicine.

[25] Anderson DG (2012). A radiographic analysis of degenerative spondylolisthesis at the L4–5 level. J. Neurosurg. Spine.

[26] Bach K (2018). Morphometric analysis of lumbar intervertebral disc height: An imaging study. World Neurosurg..

[27] Takahashi Y (2019). Effect of segmental lordosis on the clinical outcomes of 2-level posterior lumbar interbody fusion for 2-level degenerative lumbar spondylolisthesis. J. Neurosurg. Spine.

[28] Kuhta M, Bošnjak K, Vengust R (2019). Failure to maintain segmental lordosis during TLIF for one-level degenerative spondylolisthesis negatively affects clinical outcome 5 years postoperatively: A prospective cohort of 57 patients. Eur. Spine J..

[29] Hsu HT, Yang SS, Chen TY (2016). The correlation between restoration of lumbar lordosis and surgical outcome in the treatment of low-grade lumbar degenerative spondylolisthesis with spinal fusion. Clin. Spine Surg..

[30] Schwab F (2012). Scoliosis Research Society-Schwab adult spinal deformity classification: A validation study. Spine.

[31] Gille O (2014). Degenerative lumbar spondylolisthesis: Cohort of 670 patients, and proposal of a new classification. Orthop. Traumatol. Surg. Res..

[32] Kepler CK (2015). Clinical and radiographic degenerative spondylolisthesis (CARDS) classification. Spine J..

[33] Hiyama A (2018). Correlation analysis of sagittal alignment and skeletal muscle mass in patients with spinal degenerative disease. Sci. Rep..

[34] Goubert D (2017). Lumbar muscle structure and function in chronic versus recurrent low back pain: A cross-sectional study. Spine J..

[35] Parkkola R, Rytökoski U, Kormano M (1993). Magnetic resonance imaging of the discs and trunk muscles in patients with chronic low back pain and healthy control subjects. Spine.

[36] Danneels LA, Vanderstraeten GG, Cambier DC, Witvrouw EE, De Cuyper HJ (2000). CT imaging of trunk muscles in chronic low back pain patients and healthy control subjects. Eur. Spine J..

[37] Wallwork TL, Stanton WR, Freke M, Hides JA (2009). The effect of chronic low back pain on size and contraction of the lumbar multifidus muscle. Man. Ther..

[38] Guo M (2019). Predictors of L4–L5 degenerative lumbar spondylolisthesis: L4 inclination angle and facet joint angle. World Neurosurg..

[39] Belavý DL, Armbrecht G, Richardson CA, Felsenberg D, Hides JA (2011). Muscle atrophy and changes in spinal morphology: Is the lumbar spine vulnerable after prolonged bed-rest?. Spine.

[40] Belavý DL, Gast U, Felsenberg D (2017). Exercise and transversus abdominis muscle atrophy after 60-d bed rest. Med. Sci. Sports Exerc..

[41] Kjaer P, Bendix T, Sorensen JS, Korsholm L, Leboeuf-Yde C (2007). Are MRI-defined fat infiltrations in the multifidus muscles associated with low back pain?. BMC Med..

[42] O’Sullivan PB, Phyty GD, Twomey LT, Allison GT (1997). Evaluation of specific stabilizing exercise in the treatment of chronic low back pain with radiologic diagnosis of spondylolysis or spondylolisthesis. Spine.

[43] Hides JA, Jull GA, Richardson CA (2001). Long-term effects of specific stabilizing exercises for first-episode low back pain. Spine.

[44] Dabbs VM, Dabbs LG (1990). Correlation between disc height narrowing and low-back pain. Spine.

[45] Akeda K, Yamada T, Inoue N, Nishimura A, Sudo A (2015). Risk factors for lumbar intervertebral disc height narrowing: A population-based longitudinal study in the elderly. BMC Musculoskelet. Disord..

[46] Shrout PE, Fleiss JL (1979). Intraclass correlations: Uses in assessing rater reliability. Psychol. Bull..

[47] Ritchie ME (2015). limma powers differential expression analyses for RNA-sequencing and microarray studies. Nucleic Acids Res.

